# Computational redesign of a fluorogen activating protein with Rosetta

**DOI:** 10.1371/journal.pcbi.1009555

**Published:** 2021-11-08

**Authors:** Nina G. Bozhanova, Joel M. Harp, Brian J. Bender, Alexey S. Gavrikov, Dmitry A. Gorbachev, Mikhail S. Baranov, Christina B. Mercado, Xuan Zhang, Konstantin A. Lukyanov, Alexander S. Mishin, Jens Meiler

**Affiliations:** 1 Department of Chemistry and Center for Structural Biology, Vanderbilt University, Nashville, Tennessee, United States of America; 2 Department of Biochemistry, School of Medicine, Vanderbilt University, Nashville, Tennessee, United States of America; 3 Department of Pharmacology and Center for Structural Biology, Vanderbilt University, Nashville, Tennessee, United States of America; 4 Institute of Bioorganic Chemistry, Russian Academy of Sciences, Moscow, Russia; 5 Pirogov Russian National Research Medical University, Moscow, Russia; 6 Institute for Drug Discovery, Leipzig University, Leipzig, Germany; San Raffaele Hospital: IRCCS Ospedale San Raffaele, ITALY

## Abstract

The use of unnatural fluorogenic molecules widely expands the pallet of available genetically encoded fluorescent imaging tools through the design of fluorogen activating proteins (FAPs). While there is already a handful of such probes available, each of them went through laborious cycles of *in vitro* screening and selection. Computational modeling approaches are evolving incredibly fast right now and are demonstrating great results in many applications, including *de novo* protein design. It suggests that the easier task of fine-tuning the fluorogen-binding properties of an already functional protein *in silico* should be readily achievable. To test this hypothesis, we used Rosetta for computational ligand docking followed by protein binding pocket redesign to further improve the previously described FAP DiB1 that is capable of binding to a BODIPY-like dye M739. Despite an inaccurate initial docking of the chromophore, the incorporated mutations nevertheless improved multiple photophysical parameters as well as the overall performance of the tag. The designed protein, DiB-RM, shows higher brightness, localization precision, and apparent photostability in protein-PAINT super-resolution imaging compared to its parental variant DiB1. Moreover, DiB-RM can be cleaved to obtain an efficient split system with enhanced performance compared to a parental DiB-split system. The possible reasons for the inaccurate ligand binding pose prediction and its consequence on the outcome of the design experiment are further discussed.

## Author summary

Computational approaches have recently made significant progress in the protein engineering field evolving from a tool for helping experimentalists to prioritize or short-list mutations for testing to being capable of making fully reliable predictions. However, not all the fields of protein modeling are evolving at a similar pace. That is why evaluating the capabilities of computational tools on different tasks is important to provide other scientists with up-to-date information on the state of the field. Here we tested the performance of Rosetta (one of the leading macromolecule modeling tools) in improving small molecule-binding proteins. We successfully redesigned a fluorogen binding protein DiB1 –a protein that binds a non-fluorescent molecule and enforces its fluorescence in the obtained complex–for improved brightness and better performance in super-resolution imaging. Our results suggest that such tasks can be already achieved without laborious library screenings. However, the flexibility of the proteins might still be underestimated during standard modeling protocols and should be closely evaluated.

## Introduction

Fluorogenic molecules are compounds whose ability to fluoresce can be modulated, for example, by a chemical modification, change in the environment, or electronic structure [1]. A number of fluorogenic molecules have been discovered in living organisms, among them are retinal, flavin mononucleotide, tetrapyrroles such as biliverdin and bilirubin, *etc*. The biological role of many natural fluorogens is often directly connected to their ability to absorb light. For example, chlorophyll is used in photosystems of cyanobacteria, algae, and plants; flavins are essential parts of DNA photolyases [2] and cryptochromes [3]; rhodopsin, covalently bound to retinal, is required for vision in many animals. The dissipation of the absorbed energy through fluorescence in these cases is usually undesirable. Less commonly, the fluorescence of natural fluorogenic molecules appears to be only a side effect or its function is not yet understood. For example, a fatty-acid-binding protein UnaG has been discovered and cloned from Japanese eel. UnaG binds bilirubin, which allows for its bright fluorescence, but the biological role of the observed fluorescence is not known [4].

A number of fluorescent probes has been created by mutating natural fluorogen-binding proteins to promote their fluorescence. For example, starting from bacterial light-oxygen-voltage–sensing domains, such an effort yielded flavin-binding fluorescent proteins [5]; different bacteriophytochromes were converted into near-infrared probes IFP1.4 [6] and iRFP [7]. Later, proteins capable of binding and increasing the fluorescent signals of fluorogenic molecules became commonly referred to as fluorogen activating proteins (FAPs).

In addition to natural fluorophores, fluorogenic compounds can be synthesized. The existing examples include but are not limited by BODIPY dyes, rhodamines, cyanines, and coumarins [1]. The utilization of these molecules for fluorescent labeling is quite tempting due to multiple reasons. That includes their high spectral and chemical diversity. These compounds can be selected for being orthogonal to the normal biological functions of investigated systems. External addition of such a ligand provides full control and flexibility over the timing of acquiring the signal.

Several FAPs have been designed so far to use unnatural fluorogenic molecules. Screening of libraries of antibodies resulted in the discovery of binders for derivatives of thiazole orange, malachite green [8], and cyanine dye dimethylindole red [9]. Directed evolution of photoactive yellow protein produced rhodanine dyes-binding proteins Y-FAST [10] and frFAST [11]. *In silico* shortlisting with the further screening of the lipocalin Blc mutants launched DiBs FAPs tags family binding green [12] and red [13] BODIPY dyes. These were further expanded using structure-based rational design [14]. Finally, a *de novo* ligand-binding β-barrel protein design using Rosetta followed by two additional rounds of modeling-directed mutagenesis of proteins selected by *in vitro* screening of a limited number of hits from the previous step yielded two DFHBI-binding proteins designated mFAP1 and mFAP2 [15].

All FAP modeling projects described above involved screening of libraries of proteins to select the one with the desired properties, which is time and resource consuming. For example, the first-generation lipocalin Blc-based FAPs (DiBs) were obtained after *in silico* modelling of more than 100 000 mutants followed by a rigid body docking of a library of green fluorescent protein (GFP) chromophore-like ligands. Nineteen mutants and ten fluorogens were shortlisted to be evaluated experimentally [12]. However, with the current progress of computational modeling tools, engineering such systems purely *in silico* might be already possible.

Here we tested the performance of one of the leading macromolecules modeling suites, Rosetta [16], on the task of fine-tuning a functional FAP protein through remodeling. We characterize a protein obtained purely by computational docking and redesign of the previously described FAP DiB1 [12] using Rosetta. Despite an inaccurate placement of the chromophore in the binding pocket, the predicted mutations nevertheless improved multiple photophysical parameters as well as the overall performance of the tag. The possible reasons for the inaccurate ligand binding pose prediction and its consequence on the outcome of the design experiment are further discussed.

## Results

### Rosetta modeling

DiB1 protein was selected as a starting point for the protein design project. Among first generation Blc-based FAPs this mutant showed the lowest *K*_d_ [12] indicating a more specific and stable binding site. The model of the DiB1 protein was generated based on the existing crystal structure of the wild type apo Blc protein (PDB ID 1QWD) [17]. For this, the identity of two amino acids in positions 36 and 141 of the wild type Blc was converted to the corresponding amino acids in DiB1, and a total of 50 structures were generated using Rosetta Relax application [18]. The best scoring model was selected for docking.

Docking of the M739 chromophore (S1 **Fig**) was performed with gradually decreasing ligand sampling freedom until the solution converged on a single binding pose (S2 **Fig**). First, 5 000 protein-ligand complexes were generated using a coarse protein binding pocket sampling strategy with 5 Å maximum ligand translation allowed per step and up to 360° rotation. The top 500 models by total energy were further sorted based on their protein-ligand interface score and the 50 best were selected for a subsequent docking round. During the next step ligand translation and rotation were restricted to 1 Å and 45°, respectively. 100 structures were generated for each of 50 starting models resulting again in a total of 5 000 output structures. 50 best structures were selected as previously and the last round of docking was performed with 0.2 Å maximum translation and 5° maximum rotation for fine-tuning of the ligand placement. The best scoring docking pose was further used for the ligand binding pocket design. In this model the ligand was located within an interaction distance from amino acids at positions 141 and 74, mutations in which were shown to influence the properties of the ligand:protein complex in our previous study [12]. While the other position that is mutated in DiB1, residue 37, locates more than 10 Å away from any of the ligand’s heavy atoms, we were not very concerned. That position was included in our initial study screening as it participates in the formation of a small pocket adjacent to the main cavity in the wild type protein. However, that pocket was formed most likely artificially as a result of the high pressure soaking with xenon used for phase calculation [17]. Therefore, it is highly probable that the amino acid in position 37 influences the protein properties in general rather than directly interacts with the ligand.

In fluorescent proteins, their ability to fluoresce and the brightness of the fluorescence highly depend on the capacity of the surrounding amino acids to stabilize the chromophore in a planar conformation (e.g. for GFP, ensuring that the phenoxy and imidazolinone moieties of the chromophore are as coplanar as possible). In the case of M739, the structure is already conformationally locked in the favorable planar conformation. Contrary, its fluorescence quantum yield (QY) was shown to vary dramatically depending on the solvent with an almost tenfold increase in dioxane compared to water [19]. Thus, having no specific protein:ligand interactions that seem important to emplace (which is usually achieved by employing protocols derived from the Rosetta enzyme design protocol [20]), we hypothesized that further improvement of DiB1 might be achieved through the refining of the protein:ligand interface packing using a conventional RosettaLigand design protocol [21] (S3 **Fig**). The protocol was previously shown to perform better on apolar small molecules whose binding is dominated by van der Waals interactions [22].

From the starting complex structure, we generated 5 000 designs. Amino acids with their Cα atoms within 6 Å of any ligand atom or with their Cα atoms within 8 Å of any ligand atom and with their Cα-Cβ vector pointing toward the ligand were allowed to be designed to any amino acid except cysteine. The residues with their Cα atoms within 10 Å of any ligand atom or with their Cα atoms within 12 Å of any ligand atom and their Cα-Cβ vector pointing toward the ligand were allowed to repack. The 2 000 best scoring designs were then sorted based on their ligand-protein interface score and the best 50 were selected for detailed analysis.

**Fig 1:**
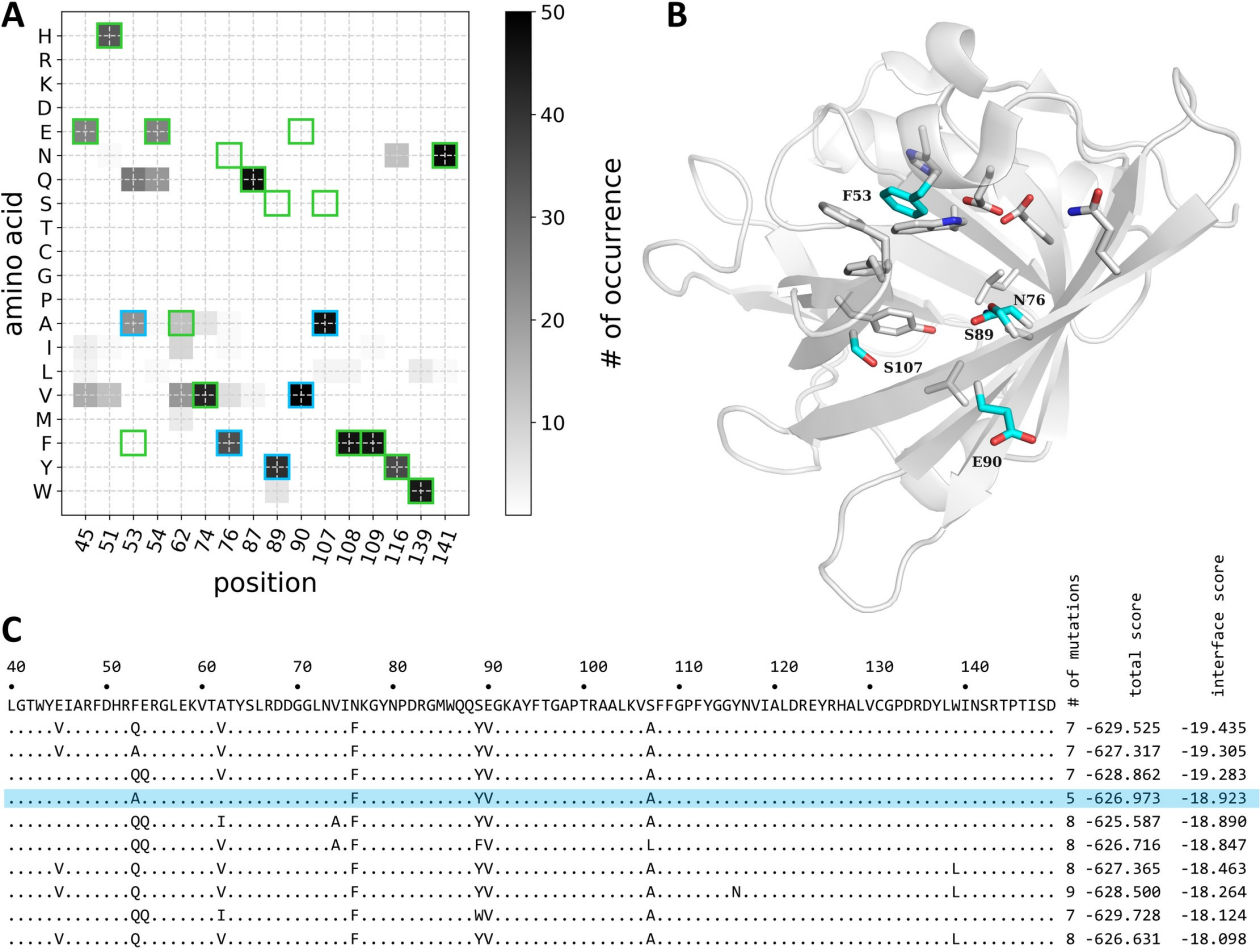
Rosetta Design results. (**A**) The summary of the mutations observed in the 50 best (sorted by interface score) models output of the Rosetta Design protocol. The frequency of observation of the given amino acid (y-axis) at the given position (x-axis) in the analyzed variants is indicated by color (light grey–less often, dark grey–more often). Amino acid identities in the parental protein DiB1 are indicated as green squares. Amino acid substitutions in the selected for experimental evaluation protein DiB-RM are indicated as cyan squares. (**B**) Cartoon representation of the lipocalin Blc (PDB ID 1QWD, chain A). The positions in which mutations were observed among the 50 best (sorted by interface score) models output of the Rosetta Design protocol are shown as sticks. The positions which were mutated in the variant selected for experimental testing (DiB-RM) are colored cyan and labeled. (**C**) A fragment of the alignment of the DiB1 amino acid sequence (top line) and ten best design sequences (sorted by interface score). There are no mutations in any of the proteins outside the shown region. Dots indicate the same amino acid in the given position as in DiB1. The sequence selected for the experimental testing (DiB-RM) is highlighted in light blue.

A total of 16 amino acid positions were found to be mutated at least in one of the selected designed models (**Fig 1A and 1B**). Mutations at positions 74, 108, 109, 116, 139, and 141 were relatively rare with the majority of the generated sequences retaining the native amino acid at those positions. In contrast, amino acids at positions 53, 76, 89, 90, and 107 were mutated in almost all 50 selected models. Four of these positions showed strong convergence toward one specific mutation: Phe at position 76, Tyr at position 89, Val at position 90, and Ala at position 107. Amino acid 53 was mutated to either alanine or glutamine with nearly equal frequency in the models.

Of note, one of the top-scoring models (**Fig 1C**) contained only mutations at these five positions. Four residues (positions 76, 89, 90, and 107) were converted to the most favorable amino acids for these positions. Alanine, the second most frequent amino acid in position 53 among 50 best-scored poses, was found in position 53 in that sequence. After additional manual examination, this sequence (designated DiB-RM) was selected for testing.

### Experimental characterization of DiB-RM

We first compared the photophysical properties of DiB-RM in complex with M739 chromophore with the parental protein DiB1 *in vitro* (**Table 1**). In comparison with DiB1, the DiB-RM:M739 complex, with almost identical fluorescence spectra, demonstrated an increase in both quantum yield (QY) and extinction coefficient (ɛ) resulting in approximately 25% increased brightness. These changes were accompanied by some loss in the apparent binding affinity of the protein to the ligand.

**Table 1:** Properties of the DiB protein–chromophore M739 complexes.

Name	λ_ex_, nm	λ_em_, nm	*K*_d_, μM	QY, %	ɛ, M^-1^cm^-1^
**DiB1**	512	543	0.04 ± 0.03	50	51 400 ± 2 300
**DiB-RM**	511	543	0.17 ± 0.04	59	56 000 ± 1 100
**DiB-RM-split**	517	548	2.8 ± 0.4	39	59 400 ± 3 700
**M739 (free ligand)**	517	564	n/a	3.5^a^	53 500^a^

n/a–not applicable

λ_ex_−wavelength of excitation spectrum maximum

λ_em_−wavelength of maximum emission intensity

^a^—data from Bozhanova et al. 2017

**Fig 2:**
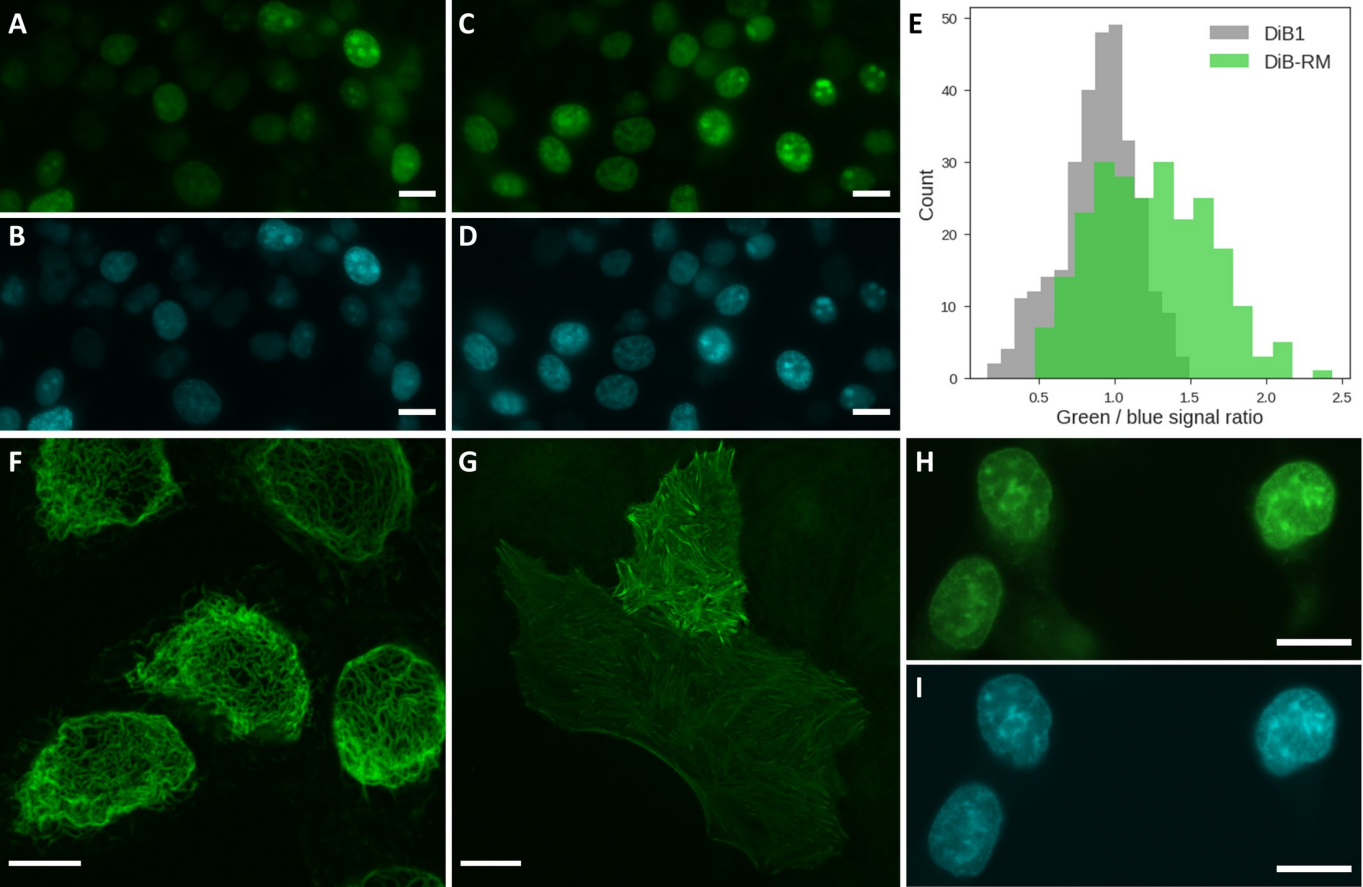
Experimental characterization of new proteins in cellulo. (**A-D**) Representative widefield fluorescence images of living HEK293 cells transiently transfected with H2B-TagBFP-DiB1 (**A, B**) or H2B-TagBFP-DiB-RM (**C, D**) constructs in presence of 0.5 μM M739 in green (**A, C**) and blue (**B, D**) detection channels; scale bars are 15 μm. (**E**) Green to blue fluorescence signal ratio distribution in nuclei of living HEK293 cells transiently transfected with H2B-DiB1-TagBFP or H2B-DiB-RM-TagBFP in presence of 0.5 μM M739. Represented are data for 307 cells transiently transfected with H2B-DiB1-TagBFP and 241 cells transiently transfected with H2B-DiB-RM-TagBFP. (**F-G**) Confocal fluorescence microscopy imaging (excitation: 488 nm, emission: 520–560 nm) of living HeLa cells transiently transfected with DiB-RM constructs in presence of 0.25 μM M739; (**F**) vimentin-DiB-RM, scale bar 10 μm; (**G**) LifeAct-TagBFP-DiB-RM, scale bar 20 μm. (**H-I**) Widefield fluorescence images of living HEK293 cells transiently cotransfected with H2B-DiB-RM-splitN_1-109_ and DiB-RM-splitC_110-177_-TagBFP constructs in presence of 0.1 μM M739 in green (**H**) and blue (**I**) detection channels; scale bars are 15 μm.

We next characterized the DiB-RM protein using transiently transfected living cells. We first generated a fusion protein of DiB-RM with histone H2B and blue fluorescent protein TagBFP, which was compared with a previously created H2B-TagBFP-DiB1 construct [12]. Using TagBFP fluorescent signal as an internal control, we compared the brightness of DiB1 and DiB-RM *in cellulo* (**Figs 2A–2E and S4**). In concordance with the results obtained *in vitro*, DiB-RM demonstrated increased brightness compared to DiB1: ~ 1.38:1 versus ~ 1.29:1 observed *in vitro*.

We next created DiB-RM fusions with various structural proteins (vimentin, actin-binding peptide LifeAct) and imaged transiently transfected mammalian cells using confocal fluorescence microscopy. The target intracellular structures were brightly fluorescent immediately upon the addition of the fluorogen and did not show any signs of mislocalization or aggregation (**Fig 2F and 2G**).

### DiB-RM-split

We previously created a self-assembling split system from the first generation of DiB proteins [23]. Despite the observed spontaneous reassembly of the DiB proteins split between amino acid residues 109 and 110 into functional proteins that recapitulated the overall structure and function of the full-length lipocalins in the case of co-expression in *E*. *coli*, we had to move the split point further to the C-terminus due to the observed severe aggregation of the N-fragment expressed alone in mammalian cells. Taken the overall better performance of the new DiB-RM FAP as well as the fact that all DiB-RM mutations are localized in the N-terminal part of the protein relative to the original split point, we examined the influence of these five introduced mutations on the performance of this new FAP as a split system. We started with the introduction of the chain break in DiB-RM in the original position (between residues 109 and 110) and tested the behavior of the fusion protein composed of the newly obtained N fragment (designated DiB-RM-splitN_1-109_) with a fluorescent protein mNeonGreen in transiently transfected HEK cells. Interestingly, we did not observe aggregation (S5A **Fig**) as was seen previously for DiB2-split N fragment in a similar experiment. Inspired by this observation we also checked free DiB-RM-split C fragment distribution in cells. There is only one amino acid difference between DiB2 and DiB-RM proteins downstream to the split point (L141N) and it did not affect the performance of the DiB-RM-split C fragment: HEK cells transiently transfected with the DiB-RM-splitC_110-177_-TagBFP construct showed uniform labeling in the blue fluorescent channel (S5B **Fig**). We then tested the self-assembly capability of the DiB-RM N and C fragments obtained via split between amino acid residues 109 and 110. Transient cotransfection of H2B-DiB-RM-splitN_1-109_ and TagBFP-DiB-RM-splitC_110-177_ constructs in living cells revealed successful attraction of TagBFP-DiB-RM-splitC_110-177_ fusion protein to the nuclei despite lacking a nuclear localization signal (**Fig 2I**) and staining nuclei in green upon the chromophore addition (**Fig 2H**) as a result of efficient self-assembly of split-DiB-RM.

We also characterized the DiB-RM-split:M739 complex *in vitro* (**Table 1**) using purified protein. As was previously observed for other self-assembling DiB-splits [23], splitting of the protein resulted in a decrease in apparent binding affinity to the chromophore compared to the full-length parental protein. However, in the case of DiB-RM, the observed decrease was more pronounced. We speculate that the introduction of three mutations with substantial side chain size difference (F53A, N76F, and S89Y) at the split interface might have changed the split protein stability and/or assembly kinetics. The introduced chain break also decreased the complex QY and slightly shifted the fluorescence excitation and emission maxima, which might also indicate higher solvent accessibility of the ligand. Contrary to other DiB-splits, DiB-RM-split demonstrated an increased extinction coefficient.

### Super-resolution microscopy

**Fig 3:**
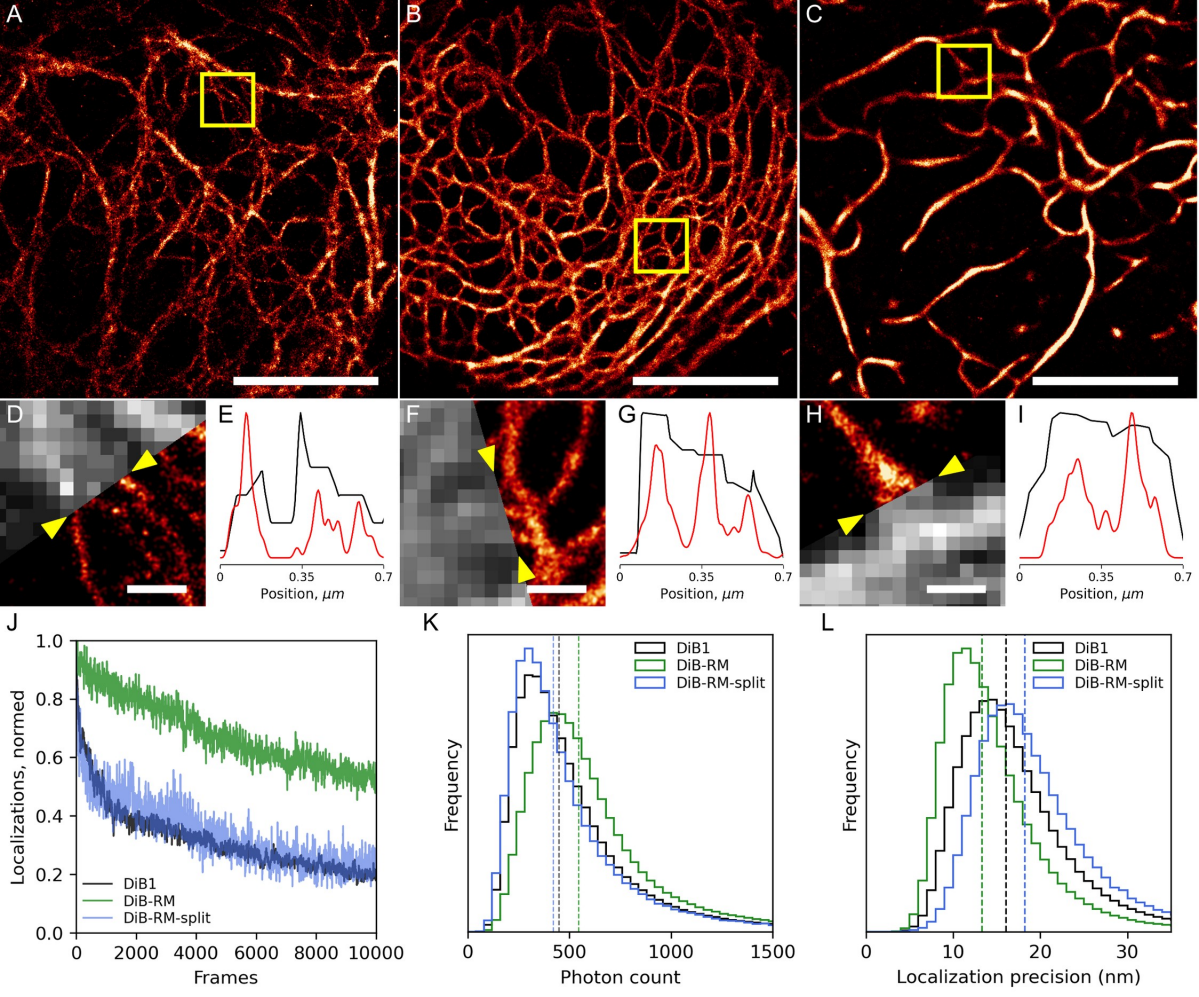
DiB1, DiB-RM, and DiB-RM-split performance in super-resolution microscopy of living cells. HeLa cells transiently transfected with vimentin-DiB1 (**A, D, E**), vimentin-DiB-RM (**B, F, G**), and transiently co-transfected with vimentin-DiB-RM-splitN_1-109_ + DiB-RM-splitC_110-177_-TagBFP constructs (**C, H, I**) in the presence of 20 nM M739. Imaging conditions: 1.1 kW cm^-2^ of 488 nm laser light, 30Hz acquisition frequency. (**A-C**) Super-resolution reconstruction from 10 000 frames, scale bars are 5 μm. (**D, F, H**) Average projection of 1 000 frames and super-resolution reconstructions from 10 000 frames; scale bars are 0.5 μm. (**E, G, I**) Normalized intensity profiles between yellow arrows shown on the images (**D, F, H**); black curve–widefield and red curve–super-resolution. (**J-L**) Comparison of DiB1, DiB-RM, and DiB-RM-split performance in a localization microscopy setup, average values for 7 cells. (**J**) Photostability in the localization microscopy setup. (**K**) The number of detected photons per single-molecule event; vertical lines represent median values. (**L**) Localization precision per single-molecule event; vertical lines represent median.

The main power of DiB FAPs lies in single-molecule localization microscopy of living cells [12,14]. We therefore performed a side-by-side comparison of the super-resolution localization microscopy performance of DiB-RM and DiB-RM-split proteins with the parental DiB1 tag (**Fig 3 and S1–S3 Movies**). All three tags provided reconstructions of vimentin fibers with better resolution than widefield fluorescence microscopy (**Fig 3A–3I**). DiB1 showed an initial exponential decrease in the number of localizations that later turned into a linear decrease down to ~20% of the initial localization count. The DiB-RM signal decreased only linearly throughout the experiment. That allows for the accumulation of a much higher number of localizations during the same time period using DiB-RM. DiB-RM-split tag performed similarly to DiB1 (**Fig 3J**). DiB-RM also demonstrated higher single-molecule brightness (median photon counts per single-molecule event equal to 540) than DiB-RM-split and DiB1 (median photon counts per single-molecule event equal to 446 and 419, respectively, **Fig 3K**). Both DiB1 and DiB-RM-split provided lower localization precision (median precision values equal to 16 and 18.2 nm respectively) compared to DiB-RM (median precision value equals to 13.3 nm, **Fig 3L**).

### Structural analysis

**Fig 4:**
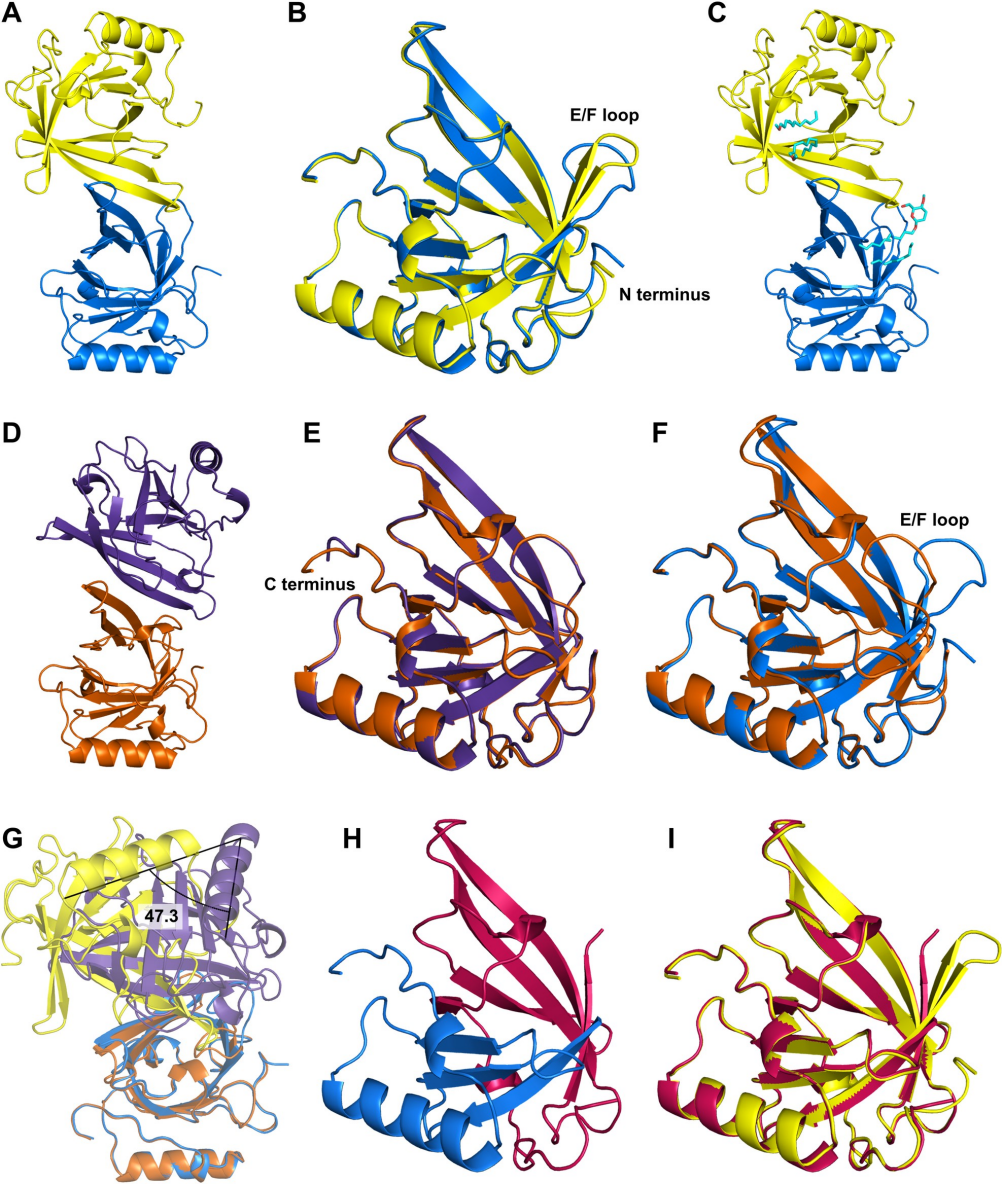
DiB-RM crystal structures analysis. Crystal structures of the full-length DiB-RM protein crystallized in the presence of DDM (**A-C**) or without it (**D-E**), and their comparison (**F-G**). Cartoon representation of the content of the asymmetric unit (**A, D**), the overlay of two protein chains from the asymmetric unit (**B, E**), and binding sites of the DDM molecules (**C**). The difference in the protein conformation between two crystals (**F**, DiB-RM protein structure obtained in the presence of DDM is colored blue, without DDM–colored orange) and in chain’s packing in the asymmetric unit (**G**). (**H**) Crystal structure of the DiB-RM-split protein. The N terminus part of the split protein is colored pink, the C terminus part of the split is colored blue. (**I**) The lipocalin fold is well preserved in DiB-RM-split protein as showed by the overlay of DiB-RM-split protein (both fragments colored pink) with the full-length DiB-RM (colored yellow).

To further characterize DiB-RM we obtained a number of crystal structures. We first successfully crystalized full-length DiB-RM in *apo* form. The asymmetric unit contains two copies of the protein with the canonical lipocalin fold, a β-barrel with an α-helix (**Fig 4A**). The two copies align well (0.77 Å rmsd across 158 Cα atoms) with the main difference found in the E/F loop and at the N terminus of the protein (**Fig 4B**).

Interestingly, during structure refinement, we discovered positive difference density map features in the binding pocket of the molecules. Based on the shape of the density we speculated that they belong to the dodecyl chains of n-Dodecyl-β-D-maltoside (DDM) present in the crystallization buffer (**Fig 4C**).

We also obtained colored crystals in similar conditions using soaking or co-crystallization of DiB-RM with M739 (S6 **Fig**). Unfortunately, we still observed the same long carbon chain-like density in the binding pocket of DiB-RM in these crystals. It might be explained by the higher affinity of DiB-RM to DDM than to M739 or by the requirement of the presence of DDM in the binding pocket of the protein for crystal formation in these conditions.

To test the former hypothesis, we assessed the binding affinity of DDM to DiB-RM using tryptophan fluorescence quenching assay. Surprisingly, we have not detected any spectral changes upon the addition of up to 50x molar access of the DDM to the protein solution in normal buffer conditions. While this experiment cannot fully reproduce the processes and their dynamics that are happening during crystallization, the requirement of the presence of DDM for crystal formation in these conditions seems to be a more likely explanation.

We later obtained other *apo* DiB-RM crystals in DDM-free conditions. This crystal also contains two protein molecules in the asymmetric unit (**Fig 4D**). However, the relative orientation of the molecules differs: we observed approximately 50 degrees rotation (**Fig 4G**). This observation along with the data from ’Protein interfaces, surfaces and assemblies’ service PISA [24] suggests that the dimerization is a crystallographic artifact and the protein exists as a monomer in solution. The two protein copies from the asymmetric unit align even better with only 0.47 Å rmsd across corresponding 156 Cα atoms defined in both chains. The C terminal residues contributed the most to the difference (**Fig 4E**).

In comparison to the first structure, the main difference was observed in the conformation of the E/F loop of the protein. In the new structure, this loop is bent inwards and almost fully closes the entrance to the ligand binding pocket of the protein (**Fig 4F**). Unsurprisingly, the crystals remained clear after the addition of the ligand in the drops.

We also obtained DiB-RM-split *apo* protein crystals with two split fragments, N terminus and C terminus, forming one “full” lipocalin molecule in the asymmetric unit (**Fig 4H**). Despite the backbone cleavage, the lipocalin fold is well preserved as it was previously observed for other DiB-derived split proteins [23] (**Fig 4I**).

Multiple colored crystals from DiB-RM-split:M739 mixture were obtained in 1.8 M lithium sulfate and 6% 2-propanol buffer in presence of different additives (S6 **Fig**). The carbon chain-like density in the binding pocket was absent, which further supports our hypothesis that the previously observed density in full-length DiB-RM crystals indeed belongs to DDM and not to some other molecule co-purified with the protein. However, we were not able to locate any density for the M739 ligand either.

## Discussion

Using a fixed backbone Rosetta Design protocol, we predicted a set of mutations to improve the first generation lipocalin Blc-based FAP DiB1. The resulting protein, designated DiB-RM, performs better than its parental variant both *in vitro* (except for the dissociation constant) and as a tag for protein-PAINT [12] with higher brightness, localization precision, and apparent photostability. We speculate that the observed decrease in affinity to the ligand is caused by introducing two aromatic amino acids in the protein’s binding pocket (76F and 89Y). While the presence of these amino acids is most likely responsible for the observed increased brightness (**Table 2**) of the DiB-RM:M739 complex compared to the parental DiB1:M739 through the better protein:ligand interface packing, in the absence of the ligand these bulky amino acids might partially hinder the entrance of the ligand in the binding pocket.

**Table 2:** Properties of the reverse single mutant variants of DiB-RM–chromophore M739 complexes.

Name	λ_ex_, nm	λ_em_, nm	*K*_d_, μM	QY, %	ɛ, M^-1^cm^-1^
**DiB-RM**	511	543	0.17 ± 0.04	59	56 000 ± 1 100
**DiB-RM-A53F**	509	542	0.16 ± 0.04	57	56 800 ± 1 800
**DiB-RM-F76N**	516	547	0.07 ± 0.01	54.5	49 900 ± 900
**DiB-RM-Y89S**	514	546	0.66 ± 0.08	52.5	53 450 ± 1 700
**DiB-RM-V90E**	511	544	0.38 ± 0.05	57	58 300 ± 1 200
**DiB-RM-A107S**	512	544	0.29 ± 0.04	55	60 350 ± 1 400

λ_ex_−wavelength of excitation spectrum maximum

λ_em_−wavelength of maximum emission intensity

Despite no intentional optimization for it, the DiB-RM-based split protein (DiB-RM-split) created analogously to the previously tested DiB2-split system [23] also behaved better. We have not observed aggregation in the separately expressed N_1-109_ fragment, and the split protein successfully assembled in living cells without the need of including the additional overlapping β strand in the C fragment. Regardless of lower brightness both *in vitro* and in super-resolution microscopy set up as well as less stable fluorescent signal compared to full-length DiB-RM tag, we speculate that the DiB-RM-split should be a scaffold of choice for further DiB-split system optimizations.

**Fig 5:**
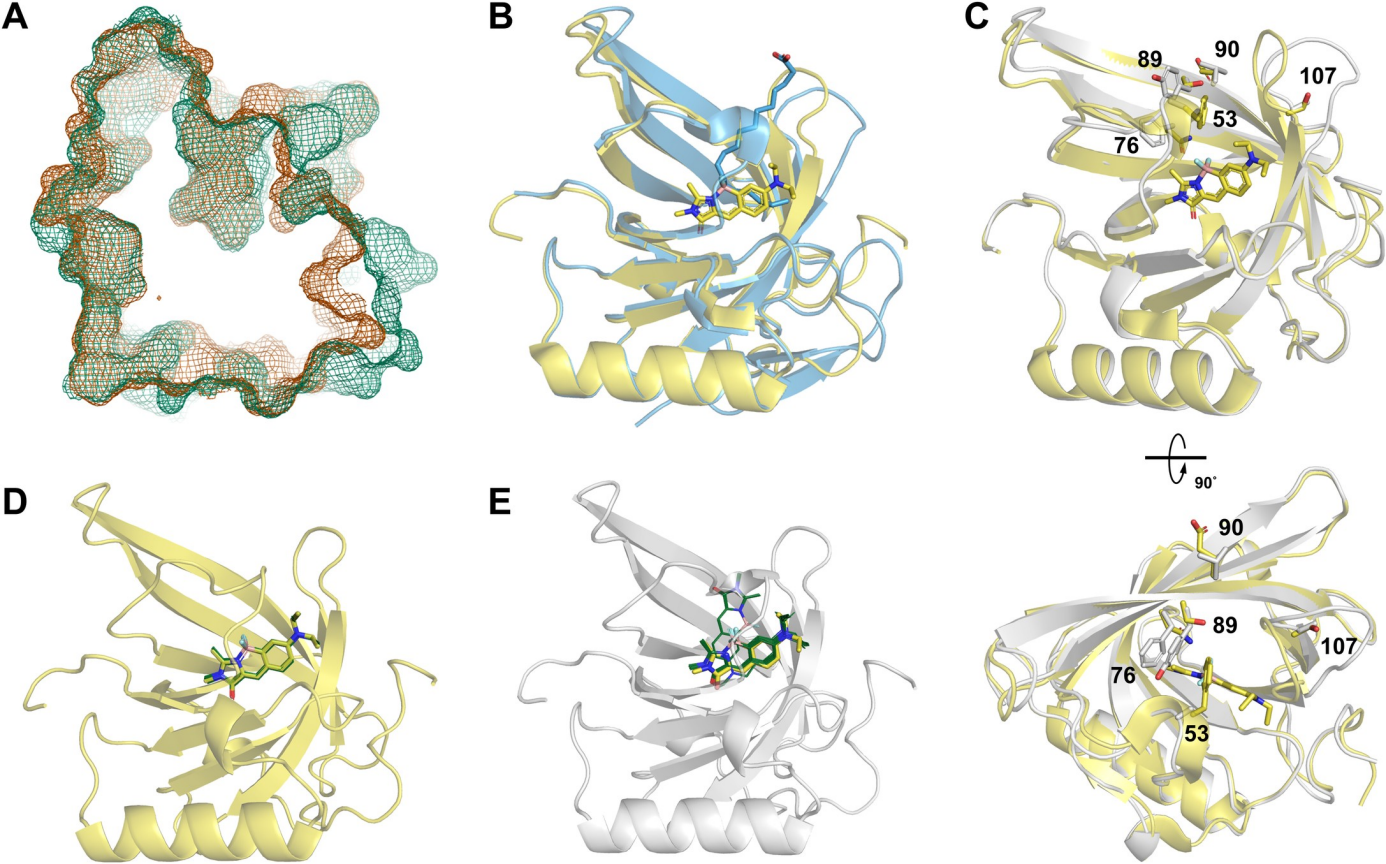
Comparison of the initial docking and modeling results with the later obtained DiB1:M739 co-crystal structure. (**A**) Binding pockets in the *apo* Blc structures showed as green (PDB ID 1QWD) or orange (PDB ID 3MBT) mesh. (**B**) Position of vaccenic acid (shown as blue sticks) and M739 (shown as yellow sticks) in the binding pocket of the lipocalin Blc (shown as a blue cartoon, PDB ID 2ACO) or DiB1 (shown as a yellow cartoon, PDB ID 6UBO), correspondingly. (**C**) The side and the top views on the overlay of the DiB1 complex (shown in yellow) with the DiB-RM *apo* structure (shown in light gray). The side chains of the amino acids in the positions that differ between DiB1 and DiB-RM as well as M739 are shown as sticks. Positions of the ligand in 50 top-scored structures after redocking of the chromophore M739 into (**D**) DiB1 crystal structure or (**E**) the DiB1 crystal structure-based DiB-RM model starting from the position of the ligand found in the DiB1:M739 co-crystal structure. The M739 chromophore from the co-crystal structure is shown as yellow sticks. All docked chromophores are shown as green lines.

Recently, the structure of DiB1 in complex with the chromophore M739 has been solved [14]. To our surprise, instead of occupying what we thought is a main binding pocket that can be seen in both *apo* Blc structures [17,25] (**Fig 5A**) and served as an entry point for the vaccenic acid that was previously co-crystallized with this lipocalin [26] (**Fig 5B**), the chromophore was found much deeper and angled (**Fig 5B**). This became possible due to the rotation of the lipocalin E/F loop. While high flexibility of this loop was previously shown [25], we failed to fully appreciated the degree of its flexibility during our initial docking experiment which resulted in a wrong ligand placement and, hence, incorporation of the mutations in the sites of the protein, which are partially not in immediate contact with the chromophore (**Fig 5C**).

Despite our inaccurate initial docking of the chromophore, it is obvious that the incorporated mutations nevertheless improved multiple photophysical parameters as well as the overall performance of the DiB tag. To further investigate this phenomenon, we ran a number of experiments.

First, to test the possibility that using a starting position of the E/F loop incompatible with the binding mode observed in the crystal structure was responsible for the failure of Rosetta docking, we reran the docking using either the DiB1 crystal structure minimized in the absence of ligand, or the same DiB1 model that was generated for the initial docking but with deleted E/F loop (-6 amino acids). In both cases, all 50 top-scored structures had a crystal-like M739 placement (**Figs 5D and S7A**) confirming that steric clashes with the backbone most likely caused our wrong initial binding mode prediction.

We then performed docking using the DiB1-based DiB-RM model generated the same way as the DiB1 model has been prepared before from the *apo* Blc structure. We tested two initial placements of the ligand: the same as the one that was initially used in our protocol and the position of the ligand in the DiB1:M739 co-crystal structure. Interestingly, in both cases, the 50 top-scored structures contained a mixture of different ligand positions one of which was matching the chromophore position in DiB1 while the other was very close to our original docked pose (**Figs 5E and S7B**). This observation together with our inability to obtain the DiB-RM:M739 co-crystal structure might indicate that we have stabilized an alternative binding site for the ligand by the introduced mutations but have not destroyed the other one. As a result, the ligand in DiB-RM might have multiple possible binding positions. This can also explain the larger apparent dissociation constant of this new complex.

To further investigate the role of the five introduced mutations we created and analyzed five reverse single mutant variants of DiB-RM (**Table 2**).

All *in vitro* characteristics of DiB-RM-A53F are almost indistinguishable from the ones of DiB-RM. Given that the side chain is solvent-exposed (S8 **Fig**), it is not surprising that this phenylalanine has been mutated by Rosetta. This substitution might be also important for the protein’s performance in the crowded cell environment.

Reintroduction of asparagine at position 76 is the only mutation that results in tighter binding to the chromophore. That might be explained by the ability of bulkier phenylalanine to sample side chain conformations that are not compatible with the ligand’s entrance to the binding pocket or its correct placement there. However, the lower *K*_d_ accompanies by the bathochromic shift of the fluorescence spectra and a decrease in both QY and ɛ. The side chain of the amino acid in position 76 packs against the ligand (S8 **Fig**). These spectral changes upon introduction of the polar residue next to the ligand align well with the effects of the polarity of the environment on the chromophore M739 properties observed in the free ligand model [19].

Reversing of the other introduced aromatic residue, DiB-RM-Y89S, results in an almost four-fold decrease in binding affinity as well as moderate spectral shifts and decrease in brightness similar to ones observed in DiB-RM-F76N mutant. The amino acid in position 89 is not in direct contact with the ligand, assuming DiB-RM interacts with the ligand similar to what is seen in the DiB1:M739 co-crystal structure (S8 **Fig**). The observed changes might support the presence of an alternative ligand binding mode in DiB-RM where Y89 packs against the ligand. Alternatively, the introduction of tyrosine at position 89 can induce more complex rearrangements in the structure of the protein. For example, through interactions with the aromatic amino acids-rich flexible E/F loop.

The remaining two variants, DiB-RM-V90E and DiB-RM-A107S, carry substitutions at the positions that are pointing outside of the ligand-binding cavity (S8 **Fig**). The most likely explanation of Rosetta’s favoring of the E90V mutation is the better β-sheet propensity of valine compared to glutamate [27]. The observed two-fold increase of *K*_d_ upon reversion of this mutation might indeed indicate its effect on the overall stability of the protein. Even less pronounced *in vitro* changes caused by A107S substitution located in a relatively flexible region of the protein (at the base of the E/F loop) make it difficult to propose its role, if any.

Overall, it appears that the majority of the mutations proposed by Rosetta were beneficial for the DiB-RM performance with the polarity of the residues in the proximity of the ligand influencing the photophysical properties of the FAP the most. Hence, refining of the protein:ligand interface using a conventional RosettaLigand design protocol seems to be a possible option for optimization of FAPs with rigid, apolar ligands. The decrease in the ligand binding affinity might be further avoided in the future by employing a multistate design protocol [28] with simultaneous optimization of the ligand binding and the stability of the ligand-binding cavity in the absence of the ligand.

Here we explored the power and limitations of Rosetta for the redesign of a protein-ligand complex. Our work resulted in the creation of an improved FAP-based fluorescent tag, however, potentially through stabilization of an alternative ligand binding site. Future optimization of DiB-RM might be focused on disabling one of the two suggested binding sites of the ligand. Crystallization analysis of DiB-RM in the *apo* form in different conditions further confirmed the already known high flexibility of the E/F lipocalin loop that was not properly addressed during our computational redesign. Rigidifying of this loop through shortening or designed interactions can provide better stabilization of the ligand in the binding pocket. This in turn can allow for expanding the compatible ligand libraries towards more flexible, conformationally unlocked chromophores [29,30]. Such ligands can dramatically increase the contrast of the probe due to the extremely low quantum yield of some of these compounds in solution.

## Methods

### Molecular cloning

Five mutations suggested by computational modeling were introduced into the previously described DiB1-pBAD bacterial expression vector [12] by self-assembling cloning [31] in three steps: 1. N76F and S107A mutations; 2. S89Y and E90V mutations (producing DiB-RM-A53F variant); 3. F53A mutation. Four other reverse single mutant variants of DiB-RM (DiB-RM-F76N, DiB-RM-Y89S, DiB-RM-V90E, and DiB-RM-A107S) were obtained using Q5 site-directed mutagenesis kit (NEB).

The DiB-RM-split vectors for bacterial protein expression were created using plasmids pMRBad-Z-CspGFP (Addgene plasmid #40730), pET11a-Z-NspGFP (Addgene plasmid #40729) [32], and the full-length DiB-RM plasmid as described before [23].

The H2B-TagBFP-DiB-RM plasmid was constructed by self-assembling cloning [31] using DiB-RM-pBAD and H2B-TagBFP (Evrogen) vectors as a template. The LifeAct-TagBFP-DiB-RM construct was further made by the standard digestion-ligation cloning approach. The TagBFP-DiB-RM coding region was cut with AgeI/NotI from the H2B-TagBFP-DiB-RM plasmid and ligated into the AgeI/NotI-digested LifeAct-Dendra2[33] plasmid.

All other plasmids for mammalian expression were assembled using Golden Gate cloning following MoClo standard [34–36]. Each transcriptional unit for mammalian expression consisted of the CMV promoter, coding sequence for the fusion protein, and the SV40 terminator. All Golden Gate cloning reactions were performed in the T4 ligase buffer (SibEnzyme, Russia) supplied with 10 U of T4 ligase, 20 U of either BsaI or BpiI (ThermoFisher, USA) and 100 ng of DNA of each DNA fragments. Golden Gate reactions were performed with the following cycling conditions: 30 cycles between 37°C and 16°C (90 sec at 37°C, 180 sec at 16°C).

The resulted constructs’ amino acid sequences are provided below. The linker sequence is underlined.

*>vimentin-DiB1*
MSTRSVSSSSYRRMFGGPGTASRPSSSRSYVTTSTRTYSLGSALRPSTSRSLYASSPGGVYATRSSAVRLRSSVPGVRLLQDSVDFSLADAINTEFKNTRTNEKVELQELNDRFANYIDKVRFLEQQNKILLAELEQLKGQGKSRLGDLYEEEMRELRRQVDQLTNDKARVEVERDNLAEDIMRLREKLQEEMLQREEAENTLQSFRQDVDNASLARLDLERKVESLQEEIAFLKKLHEEEIQELQAQIQEQHVQIDVDVSKPDLTAALRDVRQQYESVAAKNLQEAEEWYKSKFADLSEAANRNNDALRQAKQESTEYRRQVQSLTCEVDALKGTNESLERQMREMEENFAVEAANYQDTIGRLQDEIQNMKEEMARHLREYQDLLNVKMALDIEIATYRKLLEGEESRISLPLPNFSSLNLRETNLDSLPLVDTHSKRTLLIKTVETRDGQVINETSQHHDDLEGDPPVATMASSPTPPRGVTVVNNFDCKRYLGTWYEIARFDHRFERGLEKVTATYSLRDDGGLNVINKGYNPDRGMWQQSEGKAYFTGAPTRAALKVSFFGPFYGGYNVIALDREYRHALVCGPDRDYLWINSRTPTISDEVKQEMLAVATREGFDVSKFIWVQQPGS*
*>vimentin-DiB-RM*
MSTRSVSSSSYRRMFGGPGTASRPSSSRSYVTTSTRTYSLGSALRPSTSRSLYASSPGGVYATRSSAVRLRSSVPGVRLLQDSVDFSLADAINTEFKNTRTNEKVELQELNDRFANYIDKVRFLEQQNKILLAELEQLKGQGKSRLGDLYEEEMRELRRQVDQLTNDKARVEVERDNLAEDIMRLREKLQEEMLQREEAENTLQSFRQDVDNASLARLDLERKVESLQEEIAFLKKLHEEEIQELQAQIQEQHVQIDVDVSKPDLTAALRDVRQQYESVAAKNLQEAEEWYKSKFADLSEAANRNNDALRQAKQESTEYRRQVQSLTCEVDALKGTNESLERQMREMEENFAVEAANYQDTIGRLQDEIQNMKEEMARHLREYQDLLNVKMALDIEIATYRKLLEGEESRISLPLPNFSSLNLRETNLDSLPLVDTHSKRTLLIKTVETRDGQVINETSQHHDDLEGDPPVATMASSPTPPRGVTVVNNFDCKRYLGTWYEIARFDHRAERGLEKVTATYSLRDDGGLNVIFKGYNPDRGMWQQYVGKAYFTGAPTRAALKVAFFGPFYGGYNVIALDREYRHALVCGPDRDYLWINSRTPTISDEVKQEMLAVATREGFDVSKFIWVQQPGS*
*>vimentin-DiB-RM-split*_*1-109*_
MSTRSVSSSSYRRMFGGPGTASRPSSSRSYVTTSTRTYSLGSALRPSTSRSLYASSPGGVYATRSSAVRLRSSVPGVRLLQDSVDFSLADAINTEFKNTRTNEKVELQELNDRFANYIDKVRFLEQQNKILLAELEQLKGQGKSRLGDLYEEEMRELRRQVDQLTNDKARVEVERDNLAEDIMRLREKLQEEMLQREEAENTLQSFRQDVDNASLARLDLERKVESLQEEIAFLKKLHEEEIQELQAQIQEQHVQIDVDVSKPDLTAALRDVRQQYESVAAKNLQEAEEWYKSKFADLSEAANRNNDALRQAKQESTEYRRQVQSLTCEVDALKGTNESLERQMREMEENFAVEAANYQDTIGRLQDEIQNMKEEMARHLREYQDLLNVKMALDIEIATYRKLLEGEESRISLPLPNFSSLNLRETNLDSLPLVDTHSKRTLLIKTVETRDGQVINETSQHHDDLEGDPPVATMASSPTPPRGVTVVNNFDCKRYLGTWYEIARFDHRAERGLEKVTATYSLRDDGGLNVIFKGYNPDRGMWQQYVGKAYFTGAPTRAALKVAFFS*
*>DiB-RM-split*_*110-177*_ *-TagBFP*
MGPFYGGYNVIALDREYRHALVCGPDRDYLWINSRTPTISDEVKQEMLAVATREGFDVSKFIWVQQPGSGDPPVATMSELIKENMHMKLYMEGTVDNHHFKCTSEGEGKPYEGTQTMRIKVVEGGPLPFAFDILATSFLYGSKTFINHTQGIPDFFKQSFPEGFTWERVTTYEDGGVLTATQDTSLQDGCLIYNVKIRGVNFTSNGPVMQKKTLGWEAFTETLYPADGGLEGRNDMALKLVGGSHLIANIKTTYRSKKPAKNLKMPGVYYVDYRLERIKEANNETYVEQHEVAVARYCDLPSKLGHKLN*
*>mNeonGreen-DiB-RM-split*_*1-109*_
MVSKGEEDNMASLPATHELHIFGSINGVDFDMVGQGTGNPNDGYEELNLKSTKGDLQFSPWILVPHIGYGFHQYLPYPDGMSPFQAAMVDGSGYQVHRTMQFEDGASLTVNYRYTYEGSHIKGEAQVKGTGFPADGPVMTNSLTAADWCRSKKTYPNDKTIISTFKWSYTTGNGKRYRSTARTTYTFAKPMAANYLKNQPMYVFRKTELKHSKTELNFKEWQKAFTDVMGMDELYKDPPVATMASSPTPPRGVTVVNNFDCKRYLGTWYEIARFDHRAERGLEKVTATYSLRDDGGLNVIFKGYNPDRGMWQQYVGKAYFTGAPTRAALKVAFFS*

The correctness of all obtained constructs was confirmed by sequencing.

### Protein expression and purification

All lipocalin proteins were expressed in XJb(DE3) Autolysis (Zymo Research) *E*. *coli* strain. Cells were grown in LB media or M9 minimal media supplemented with 100 μg/mL ampicillin (full-length DiB proteins) or 100 μg/mL ampicillin and 50 μg/mL kanamycin (split protein) at 37°C. Expression was induced by addition 0.04% L-arabinose (full-length DiB proteins) or 0.2% L-arabinose and 10 μM IPTG (split protein) at 0.8 OD. Cells were harvested after 3 hours of expression in LB or after overnight expression in minimal media at 37°C and were resuspended in PBS buffer, pH 7.4. Suspensions were frozen at -80°C and thawed at room temperature three times. DNA was destroyed by short sonication and the lysates were centrifuged to obtain cell-free extracts.

Fluorescent protein Venus was expressed in BL21(DE3) *E*. *coli* strain. Cells were grown in LB media supplemented with 100 μg/mL ampicillin at 37°C. Expression was induced by the addition of 500 μM IPTG at 0.8 OD. Cells were harvested after overnight expression at 18°C. Before purification cells were resuspended in PBS buffer, pH 7.4, and sonicated on ice. The lysates were centrifuged to obtain cell-free extracts.

The proteins were first purified using gravity flow columns with TALON metal affinity resin (Clontech) and further purified by size-exclusion chromatography on a HiLoad 16/600 Superdex 75 pg column (GE Healthcare) pre-equilibrated with 50 mM sodium phosphate buffer, pH 6.0.

### Protein concentration calculation

Protein concentrations were estimated using the Bradford dye-binding method-based [37] colorimetric assay (Bio-Rad) and bovine serum albumin standard. Single point absorption measurements (595 nm) were performed using FlexStation 3 microplate reader (Molecular Devices). All measurements were performed in triplicate.

### Chromophore binding analysis

Titrations were performed and analyzed as previously described [12] using FlexStation 3 microplate reader (Molecular Devices). In brief, a constant amount of the chromophore solution (1 μM) was added to protein solutions of different concentrations. The full fluorescence emission spectra (510–650 nm) were collected using a 490 nm excitation wavelength. Fluorescence intensity at complex emission spectrum maximum wavelength was extracted and used to determine apparent dissociation constants (*K*_d_). For each protein, the measurements were performed using at least two independent protein purifications and at least three technical replicates for each protein sample.

### Fluorescence spectra detection

Horiba Jobin Yvon Fluoromax-3 fluorometer was used to detect full fluorescence excitation and fluorescence emission spectra for excitation/emission maxima evaluation.

### Quantum yield calculations

Fluorescence quantum yield (QY) was measured relative to the fluorescent protein Venus [38]. First, the full absorbance spectra (200–600 nm, Shimadzu UV-1800 UV/Vis spectrophotometer) and fluorescence emission spectra (excitation 510 nm, emission 514–650 nm, Horiba Jobin Yvon Fluoromax-3 fluorometer) were recorded for a number of Venus protein dilutions keeping all instrumental conditions identical. Solutions with absorption at 510 nm in a range of 0.04–0.16 AU showed a good linear correlation between absorption at 510 nm and the area under the corresponding fluorescence emission curves. Second, the M739 concentration range that gives absorption at 510 nm in the range determined in the first experiment has been calculated and experimentally confirmed. Finally, the absorbance and fluorescent spectra were detected for the FAP solutions in *apo* form and in the presence of ~0.5–3.0 μM of the M739 chromophore. FAP concentrations for experiments were chosen individually for each protein based on the previously calculated *K*_d_ values to ensure that at least 95% of the added chromophore is bound to the protein (10 μM for DiB1 and DiB-RM-F76N; 10–20 μM for DiB-RM and DiB-RM-A53F; 40 μM for DiB-RM-Y89S, DiB-RM-V90E, and DiB-RM-A107S; and 40–50 μM for DiB-RM-split). Spectra of the corresponding *apo* FAP solutions were subtracted from the absorption spectra of the protein-fluorogen complexes. The absorption at 510 nm values from these corrected spectra was also plotted against the area under the corresponding fluorescence emission curves and the linear approximation of the correlation has been calculated. The QYs were then calculated as a ratio of the slopes of the protein of interest and standard curves multiplied by standard’s QY. For each protein, the measurements were performed using protein aliquots from at least two independent protein purifications. Reported is a mean value.

### Extinction coefficient calculations

Absorption spectra collected for QY calculations were also used for excitation coefficients calculations. For each of the FAP-fluorogen complexes, the complex absorption maximum has been determined. Free M739 chromophore spectra were used to define free chromophore contribution to absorption at the given wavelength and to calculated chromophore concentrations. The FAP-fluorogen complexes’ extinction coefficients were calculated using the following equation:

$$\varepsilon_{FAP}=\frac{A_{FAP}-(1-\alpha )\times A_{M739}}{\alpha \times c_{M739}}$$

where *ε_FAP_* is the FAP-fluorogen complex extinction coefficient, *A_FAP_*–the FAP-fluorogen complex absorption at maximum, *A*_*M*739_–free chromophore absorption at the FAP-fluorogen complex absorption maximum, *c*_*M*739_–total added chromophore concentration, and *α*–a fraction of added chromophore that is bound to the protein calculated based on the previously determined FAP-fluorogen complex *K*_d_s. Reported is a mean value ± s.d. of measurements obtained for at least two independently expressed and purified protein samples using at least five data points per sample.

### Crystallization, data collection, and structure determination

Full-length *apo* DiB-RM (12 mg/mL in 50 mM sodium phosphate buffer, pH 6.0, LB-expressed) was crystalized at 21°C in 2 M ammonium sulphate, 2.5% 2-propanol supplemented with 5% w/v n-Dodecyl-b-D-maltoside according to the Hampton Research Additive Screen protocol using hanging drop vapor diffusion technique. Crystals grew within 1–3 days.

Crystals of different morphology of full-length *apo* DiB-RM (12 mg/mL in 50 mM sodium phosphate buffer, pH 6.0, expressed in M9 minimal media) were also obtained at 21°C in 1.5 M lithium sulfate, 0.1 M tris hydrochloride pH 7.0, and 5% 2-propanol, supplemented with 200 mM cesium chloride using hanging drop vapor diffusion technique. For this 2 μL of protein solution were mixed with 1.2 μL of crystallization buffer (1.5 M lithium sulfate, 0.1M tris hydrochloride pH 7.0, and 5% 2-propanol) and 0.8 μL of 1 M cesium chloride from Hampton Research Additive Screen. Crystals grew within 2–3 weeks.

DiB-RM-split (12 mg/mL in 50 mM sodium phosphate buffer, pH 6.0) *apo* crystals were obtained at 21°C in 1.6 M ammonium sulfate, 0.1 M MES, pH 4.5 supplemented with 5% w/v n-Dodecyl-b-D-maltoside according to the Hampton Research Additive Screen protocol using hanging drop vapor diffusion technique. Crystals grew within 1 week.

All crystals were flash-frozen in liquid nitrogen using Parabar 10312 oil as cryoprotectant.

Diffraction data were collected at the Life Sciences Collaborative Access Team beamline 21-ID-G or 21-ID-F at the Advanced Photon Source, Argonne National Laboratory. The diffraction data were processed using the xia2 software suite [39]. The crystal structures were solved by molecular replacement with MOLREP [40] using the wtBlc structure (PDB ID 1QWD) as a search model. Model building and iterative refinement was performed with Coot [41] and REFMAC [42], respectively. The final statistics of the structures are shown in S1 **Table.** The models have been deposited into the Protein Data Bank (PDB IDs 7L5K, 7L5L, and 7L5M). Structure figures were prepared using PyMol (v.2.2.3, Schrodinger, LLC).

### Cell culture and transient transfection

HEK293 and HeLa Kyoto cells were grown in Dulbecco’s modification of Eagle’s medium (DMEM) (PanEco) supplied with 50 U/ml penicillin and 50 μg/ml streptomycin (PanEco), 2 mM L-glutamine (PanEco), and 10% fetal bovine serum (HyClone, Thermo Scientific) at 37°C and 5% CO_2_. For transient transfections, FuGENE HD reagent (Promega) was used. Immediately before imaging DMEM was replaced with HHBS media (Hanks Buffer (PanEco) supplemented with 20 mM HEPES (Sigma)).

### Fluorescence microscopy

Widefield fluorescence microscopy was performed with the Leica DMI6000B inverted microscope equipped with HC PL Apo 100x NA 1.40 oil lens and HC PL Apo 40x NA 0.85 lens, CoolLED pE-300 light source, Zyla 5.5 sCMOS camera (Andor), using GFP and BFP filter sets.

Confocal imaging was performed using an inverted Leica confocal microscope DMIRE2 TCS SP2 (Leica, Wetzlar, Germany) equipped with HCX PL APO lbd.BL 63.0x NA 1.40 OIL objective, excitation by 488 nm laser line (100 μW).

Single-molecule localization super-resolution imaging of living cells was performed with Nanoimager S (ONI, UK) microscope at 37°C. The microscope was equipped with Olympus UPlanSApo 100x NA 1.40 oil immersion objective. Imaging experiments performed with 1.1 kW cm^-2^ of 488 nm laser light, 33 ms frame exposure time, for 10,000 frames.

### Computational modeling

All Rosetta runs were performed with weekly release 2015.12.57698.

#### DiB1 modeling

The A36C and L141N mutations were manually introduced into the crystal structure (PDB ID 1QWD). The structure was further minimized using Rosetta Relax application [18] with the following flags:

-flip_HNQ

-no_optH false

-relax:constrain_relax_to_start_coords

-relax:ramp_constraints false

-nstruct 50

-ex1

-ex2

-use_input_sc

#### Ligand preparation

The M739 chromophore geometry was optimized by the density functional method RB3LYP, using the 6–311+G** basis set, a restricted hybrid HF-DFT SCF calculation was performed using Pulay DIIS + Geometric Direct Minimization to get a set of ideal bond lengths and angles. The conformers library for the ligand was further generated using BCL::Conf conformer generator [43].

#### Ligand docking

Docking of the M739 chromophore was performed in three steps. First, 5 000 structures were generated using 5 Å maximum ligand translation allowed per step and up to 360° rotation. 50 structures were selected for the next docking round during which ligand translation and rotation were restricted to 1 Å and 45° correspondingly. 100 structures were generated for each of the 50 starting models. Analogously 50 best structures from the second docking step were selected and the last round of docking was performed with 0.2 Å maximum translation and 5° maximum rotation.

The following RosettaScripts protocol has been used with the appropriate changes introduced to the Transform Mover during the second and the third docking rounds:

<ROSETTASCRIPTS>

<SCOREFXNS>

    <ligand_soft_rep weights = ligand_soft_rep>

        <Reweight scoretype = fa_elec weight = 0.42/>

        <Reweight scoretype = hbond_bb_sc weight = 1.3/>

        <Reweight scoretype = hbond_sc weight = 1.3/>

        <Reweight scoretype = rama weight = 0.2/>

    </ligand_soft_rep>

    <hard_rep weights = ligandprime>

        <Reweight scoretype = fa_intra_rep weight = 0.004/>

        <Reweight scoretype = fa_elec weight = 0.42/>

        <Reweight scoretype = hbond_bb_sc weight = 1.3/>

        <Reweight scoretype = hbond_sc weight = 1.3/>

        <Reweight scoretype = rama weight = 0.2/>

    </hard_rep>

</SCOREFXNS>

<SCORINGGRIDS ligand_chain = "X" width = "16">

    <vdw grid_type = "ClassicGrid" weight = "1.0"/>

</SCORINGGRIDS>

<LIGAND_AREAS>

    <docking_sidechain chain = X cutoff = 6.0 add_nbr_radius = true all_atom_mode = false minimize_ligand = 10/>

    <final_sidechain chain = X cutoff = 6.0 add_nbr_radius = true all_atom_mode = false/>

    <final_backbone chain = X cutoff = 7.0 add_nbr_radius = false all_atom_mode = true Calpha_restraints = 0.3/>

</LIGAND_AREAS>

<INTERFACE_BUILDERS>

    <side_chain_for_docking ligand_areas = docking_sidechain/>

    <side_chain_for_final ligand_areas = final_sidechain/>

    <backbone ligand_areas = final_backbone extension_window = 3/>

</INTERFACE_BUILDERS>

<MOVEMAP_BUILDERS>

    <docking sc_interface = side_chain_for_docking minimize_water = true/>

    <final sc_interface = side_chain_for_final bb_interface = backbone minimize_water = true/>

</MOVEMAP_BUILDERS>

<MOVERS>

    <Transform name = "transform" chain = "X" box_size = "8.0" move_distance = "5.0" angle = "360" cycles = "5000" temperature = "5" initial_perturb = "1.0"/>

    <HighResDocker name = high_res_docker cycles = 1 repack_every_Nth = 1 scorefxn = ligand_soft_rep movemap_builder = docking/>

    <FinalMinimizer name = final scorefxn = hard_rep movemap_builder = final/>

    <InterfaceScoreCalculator name = add_scores chains = X scorefxn = hard_rep/>

</MOVERS>

<PROTOCOLS>

    <Add mover_name = transform/>

    <Add mover_name = high_res_docker/>

    <Add mover_name = final/>

    <Add mover_name = add_scores/>

</PROTOCOLS>

</ROSETTASCRIPTS>

#### Protein design

The following RosettaScripts protocol has been used to for DiB1 redesign:

<ROSETTASCRIPTS>

<SCOREFXNS>

    <ligand_soft_rep weights = ligand_soft_rep>

        <Reweight scoretype = fa_elec weight = 0.42/>

        <Reweight scoretype = hbond_bb_sc weight = 1.3/>

        <Reweight scoretype = hbond_sc weight = 1.3/>

        <Reweight scoretype = rama weight = 0.2/>

    </ligand_soft_rep>

    <hard_rep weights = ligandprime>

        <Reweight scoretype = fa_intra_rep weight = 0.004/>

        <Reweight scoretype = fa_elec weight = 0.42/>

        <Reweight scoretype = hbond_bb_sc weight = 1.3/>

        <Reweight scoretype = hbond_sc weight = 1.3/>

        <Reweight scoretype = rama weight = 0.2/>

    </hard_rep>

</SCOREFXNS>

<SCORINGGRIDS ligand_chain = "X" width = "16">

    <vdw grid_type = "ClassicGrid" weight = "1.0"/>

</SCORINGGRIDS>

<TASKOPERATIONS>

    <DetectProteinLigandInterface name = design_interface cut1 = 6.0 cut2 = 8.0 cut3 = 10.0 cut4 = 12.0 design = 1/>

</TASKOPERATIONS>

<LIGAND_AREAS>

    <docking_sidechain chain = X cutoff = 6.0 add_nbr_radius = true all_atom_mode = false minimize_ligand = 10/>

    <final_sidechain chain = X cutoff = 6.0 add_nbr_radius = true all_atom_mode = false/>

    <final_backbone chain = X cutoff = 7.0 add_nbr_radius = false all_atom_mode = true Calpha_restraints = 0.3/>

</LIGAND_AREAS>

<INTERFACE_BUILDERS>

    <side_chain_for_docking ligand_areas = docking_sidechain/>

    <side_chain_for_final ligand_areas = final_sidechain/>

    <backbone ligand_areas = final_backbone extension_window = 3/>

</INTERFACE_BUILDERS>

<MOVEMAP_BUILDERS>

    <docking sc_interface = side_chain_for_docking minimize_water = true/>

    <final sc_interface = side_chain_for_final bb_interface = backbone minimize_water = true/>

</MOVEMAP_BUILDERS>

<MOVERS>

    <Transform name = "transform" chain = "X" box_size = "8.0" move_distance = "5.0" angle = "360" cycles = "5000" temperature = "5" initial_perturb = "1.0"/>

    <HighResDocker name = high_res_docker cycles = 1 repack_every_Nth = 1 scorefxn = ligand_soft_rep movemap_builder = docking/>

    <FinalMinimizer name = final scorefxn = hard_rep movemap_builder = final/>

    <InterfaceScoreCalculator name = add_scores chains = X scorefxn = hard_rep/>

    <FavorNativeResidue name = favor_native bonus = 1.0/>

    <ddG name = calculateDDG jump = 1 per_residue_ddg = 1 scorefxn = hard_rep/>

    <PackRotamersMover name = designinterface scorefxn = hard_rep task_operations = design_interface/>

</MOVERS>

<PROTOCOLS>

    <Add mover_name = transform/>

    <Add mover_name = favor_native/>

    <Add mover_name = designinterface/>

    <Add mover_name = high_res_docker/>

    <Add mover_name = final/>

    <Add mover_name = calculateDDG/>

    <Add mover_name = add_scores/>

</PROTOCOLS>

</ROSETTASCRIPTS>

## Supporting information

S1 Table Data Collection and Refinement Statistics. (XLSX)

S1 Fig M739 chromophore structure. (TIF)

S2 Fig Docking of the M739 chromophore into the DiB1 model using Rosetta. Each datapoint corresponds to one of 5 000 models generated for each round of the docking. Some outliers with high total and/or interface scores are not shown for better visibility of the majority of the data. Insets show overlays of the ligand positions in the 50 best models of the corresponding round of docking (points enclosed in the dashed rectangles, selected as 50 structures with the best protein-ligand interface Rosetta score among top 10% of all obtained docking poses ranked by the Rosetta total score). After the third round, docking converged on a single binding pose solution (**C**).
(TIF)

S3 Fig Outline of the employed RosettaLigand design protocol. The protocol starts with a small ligand position perturbation followed by optimization of the protein:ligand interface. Amino acids are allowed to change their identity only if it results in a significant decrease in energy, defined through a “favor native” bonus (1 REU in the used protocol). The sequence design step is followed by reevaluation of the ligand position using high resolution docking protocol. The final model is scored and saved for further analysis.
(TIF)

S4 Fig Green to blue fluorescence signal ratio in nuclei of living HeLa Kyoto cells transiently transfected with H2B-DiB1-TagBFP or H2B-DiB-RM-TagBFP in presence of different concentrations of M739. Represented are data for 35–40 cells from the same field of view across the whole ligand concentration range. Box whiskers indicate standard deviation, horizontal lines within boxes indicate median values, circles indicate outliers.
(TIF)

S5 Fig Widefield fluorescence images of HEK293 cells transiently transfected with (A) NeonGreen-DiB-RM-splitN_1-109_ or (B) DiB-RM-split_110-177_-TagBFP constructs. The signal from green and blue channels, correspondingly. Scale bars are 25 μm.
(TIF)

S6 Fig Colored crystals of DiB-RM and DiB-RM-split protein grown in the presence of the M739 ligand. (TIF)

S7 Fig Position of the ligand in 50 top-scored structures. Rerun of the chromophore M739 docking (**A**) using the same DiB1 model that was generated for the initial docking but with deleted E/F loop (-6 amino acids) or (**B**) using the DiB1 crystal structure-based DiB-RM model starting from the initial (“old”) starting position of the ligand. The M739 chromophore from the co-crystal structure is shown as yellow sticks. All docked chromophores are shown as green lines.
(TIF)

S8 Fig Relative positioning of the introduced DiB-RM mutations to M739 assuming DiB1:M739-like binding to the ligand. (TIF)

S1 Movie Movie showing 1000 frames of a live HeLa cell transiently transfected with vimentin-DiB1 construct in the presence of 20nM M739 imaged under super-resolution conditions. Plays at 30 fps (acquisition speed 30 Hz).
(MP4)

S2 Movie Movie showing 1000 frames of a live HeLa cell transiently transfected with vimentin-DiB-RM construct in the presence of 20nM M739 imaged under super-resolution conditions. Plays at 30 fps (acquisition speed 30 Hz).
(MP4)

S3 Movie Movie showing 1000 frames of a live HeLa cell transiently co-transfected with vimentin-DiB-RM-split_1-109_ + DiB-RM-split_110-177_-TagBFP constructs in the presence of 20nM M739 imaged under super-resolution conditions. Plays at 30 fps (acquisition speed 30 Hz).
(MP4)
