# Supplementary figures and images for: Computational redesign of a fluorogen activating protein with Rosetta
Source: PLoS Comput Biol. 2021 Nov 8;17(11):e1009555. doi: 10.1371/journal.pcbi.1009555 (PMC8601599; doi:10.1371/journal.pcbi.1009555)

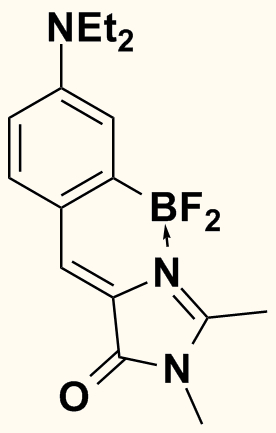

Supplement: S1 Fig — (TIF) [file pcbi.1009555.s002.tif]

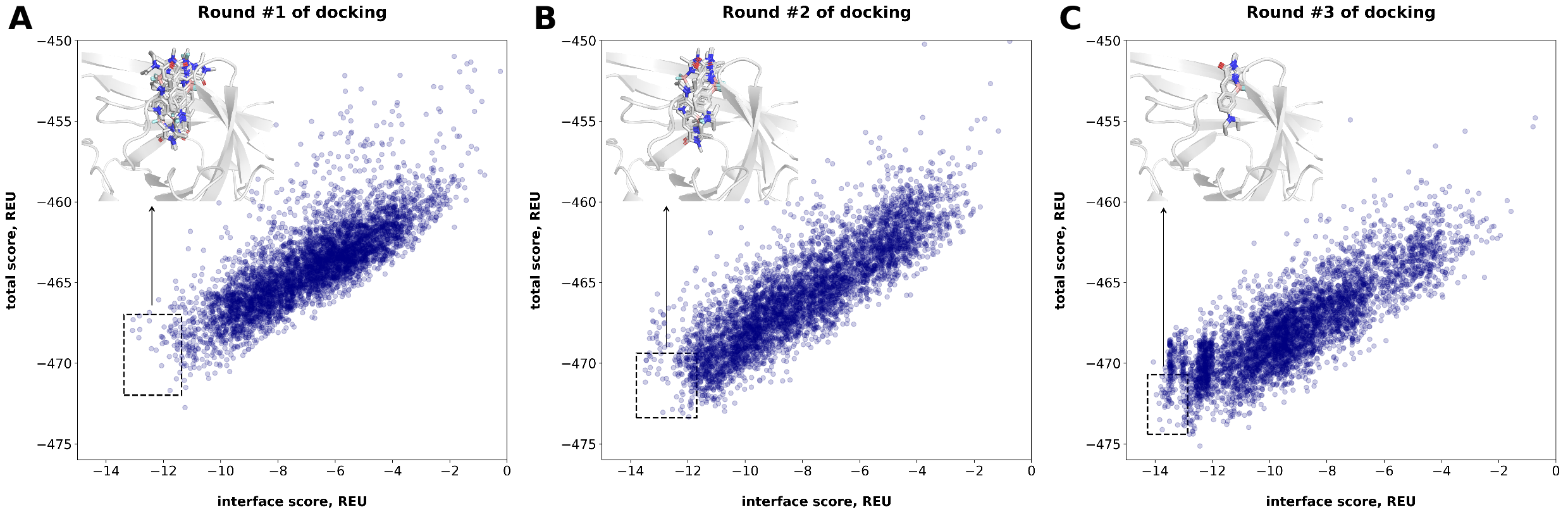

Supplement: S2 Fig — Each datapoint corresponds to one of 5 000 models generated for each round of the docking. Some outliers with high total and/or interface scores are not shown for better visibility of the majority of the data. Insets show overlays of the ligand positions in the 50 best models of the corresponding round of docking (points enclosed in the dashed rectangles, selected as 50 structures with the best protein-ligand interface Rosetta score among top 10% of all obtained docking poses ranked by the Rosetta total score). After the third round, docking converged on a single binding pose solution (C). (TIF) [file pcbi.1009555.s003.tif]

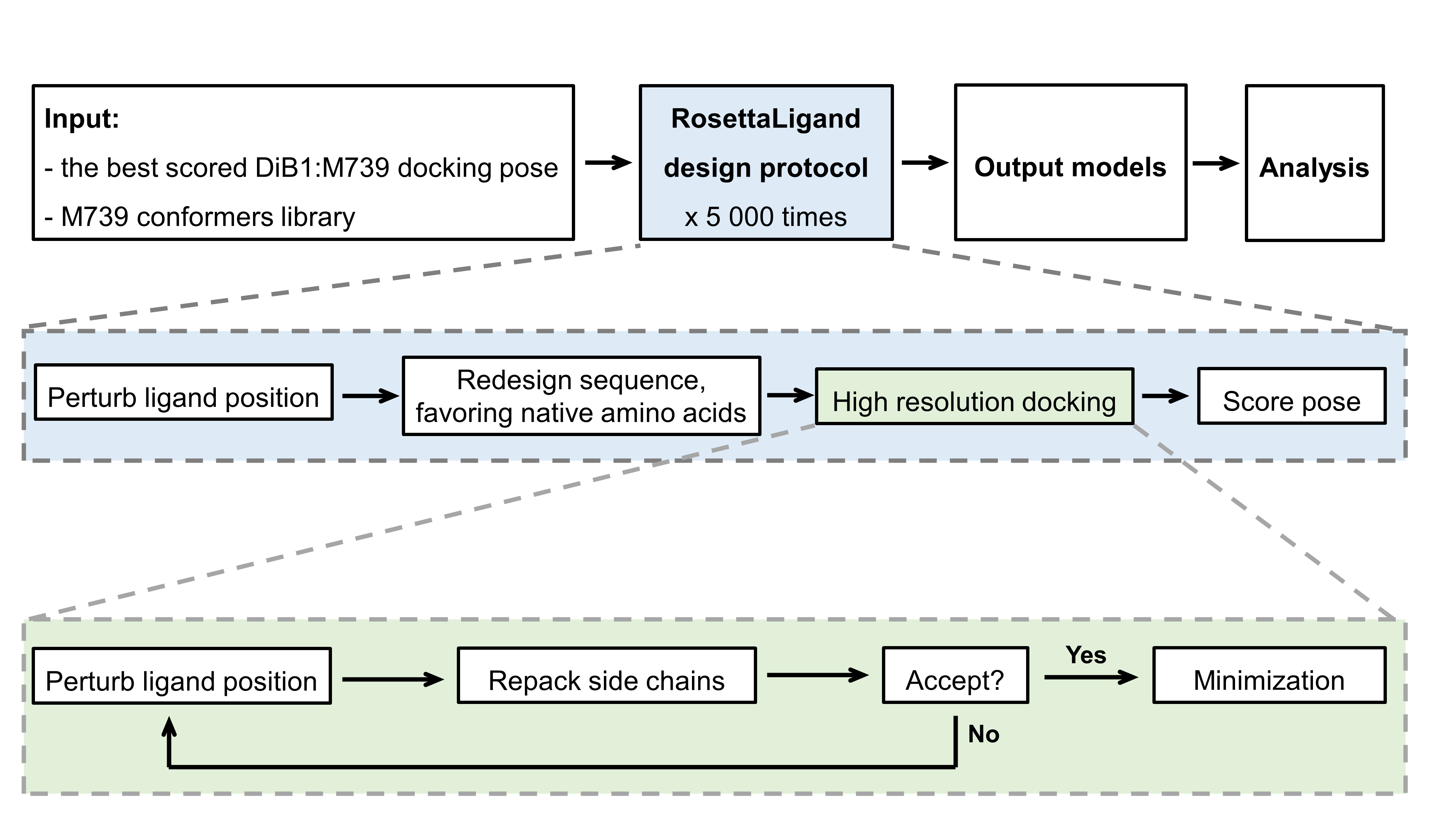

Supplement: S3 Fig — The protocol starts with a small ligand position perturbation followed by optimization of the protein:ligand interface. Amino acids are allowed to change their identity only if it results in a significant decrease in energy, defined through a “favor native” bonus (1 REU in the used protocol). The sequence design step is followed by reevaluation of the ligand position using high resolution docking protocol. The final model is scored and saved for further analysis. (TIF) [file pcbi.1009555.s004.tif]

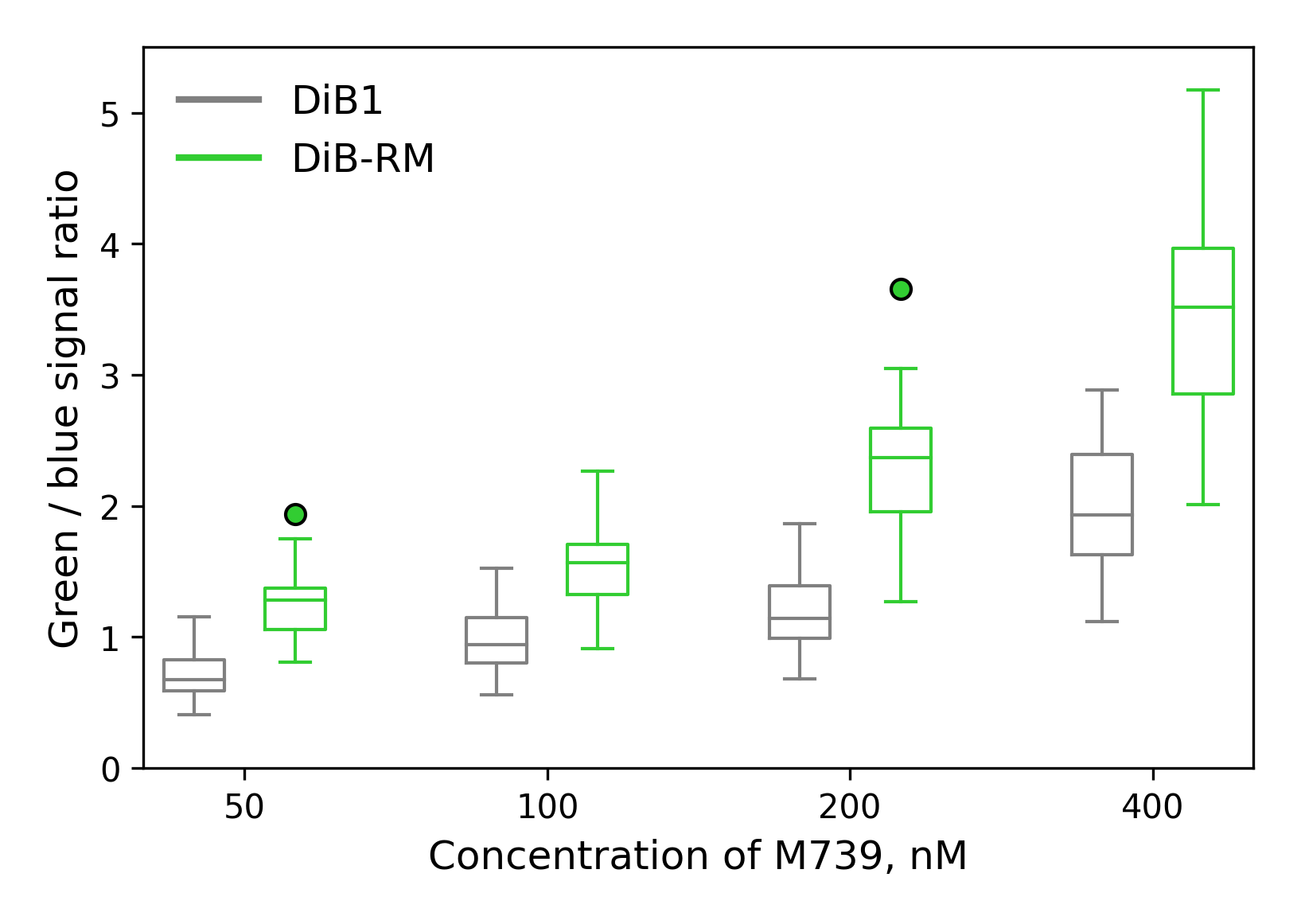

Supplement: S4 Fig — Represented are data for 35–40 cells from the same field of view across the whole ligand concentration range. Box whiskers indicate standard deviation, horizontal lines within boxes indicate median values, circles indicate outliers. (TIF) [file pcbi.1009555.s005.tif]

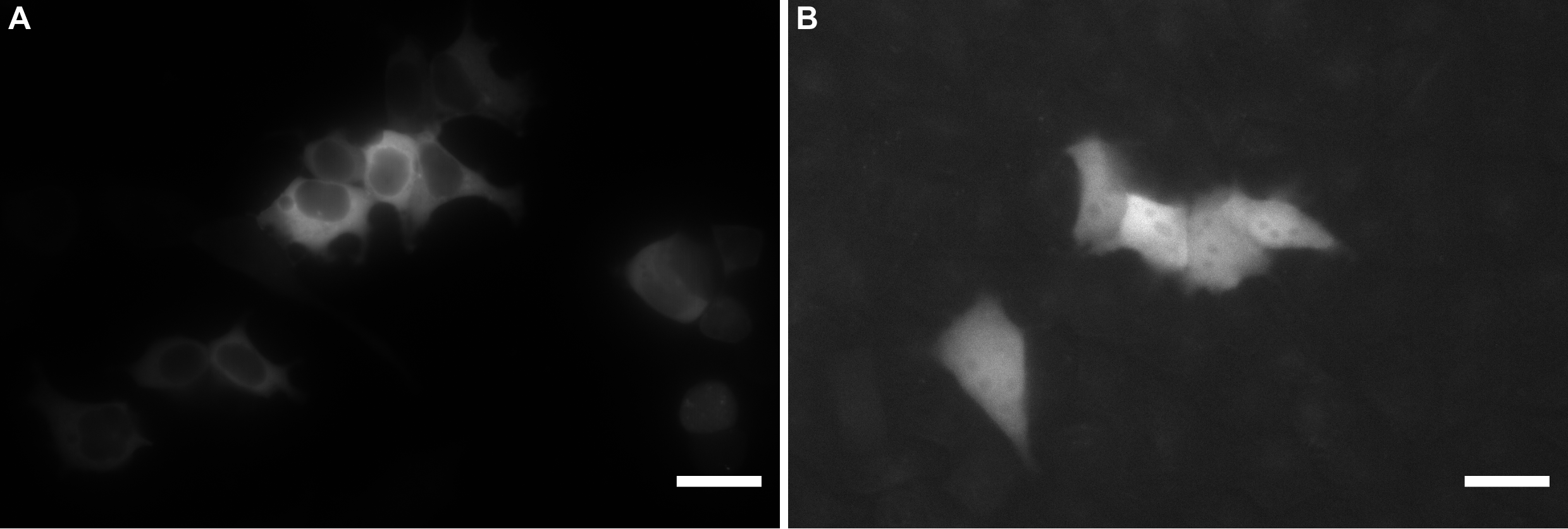

Supplement: S5 Fig — Widefield fluorescence images of HEK293 cells transiently transfected with (A) NeonGreen-DiB-RM-splitN1-109 or (B) DiB-RM-split110-177-TagBFP constructs. The signal from green and blue channels, correspondingly. Scale bars are 25 μm. (TIF) [file pcbi.1009555.s006.tif]

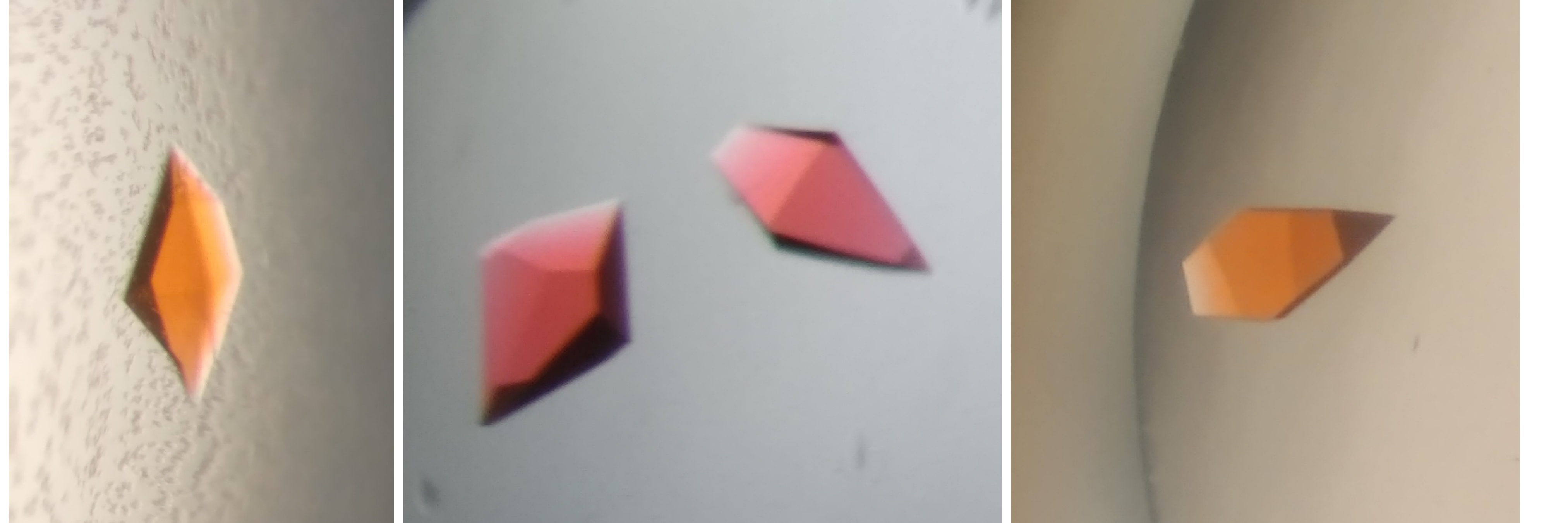

Supplement: S6 Fig — (TIF) [file pcbi.1009555.s007.tif]

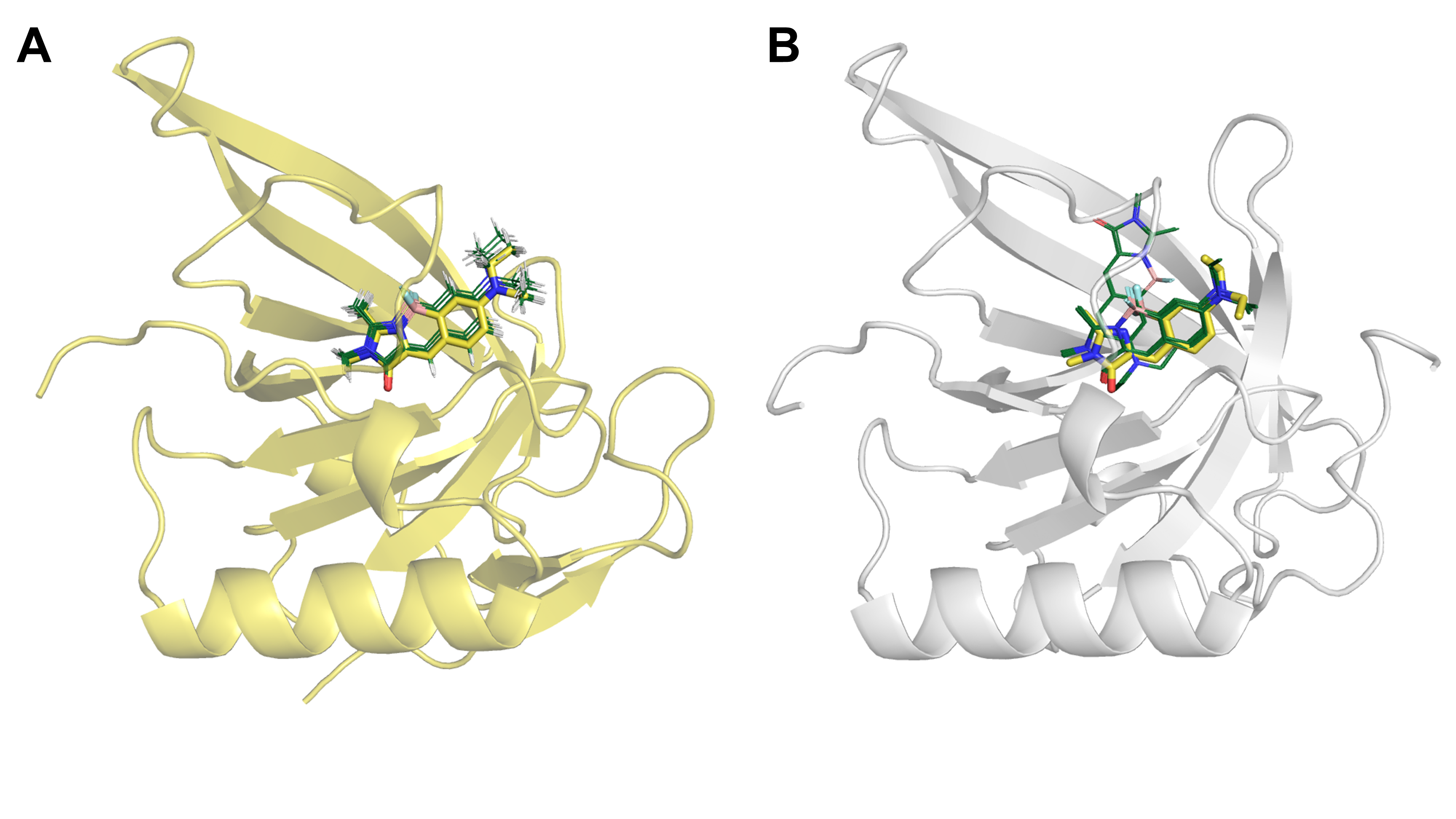

Supplement: S7 Fig — Rerun of the chromophore M739 docking (A) using the same DiB1 model that was generated for the initial docking but with deleted E/F loop (-6 amino acids) or (B) using the DiB1 crystal structure-based DiB-RM model starting from the initial (“old”) starting position of the ligand. The M739 chromophore from the co-crystal structure is shown as yellow sticks. All docked chromophores are shown as green lines. (TIF) [file pcbi.1009555.s008.tif]

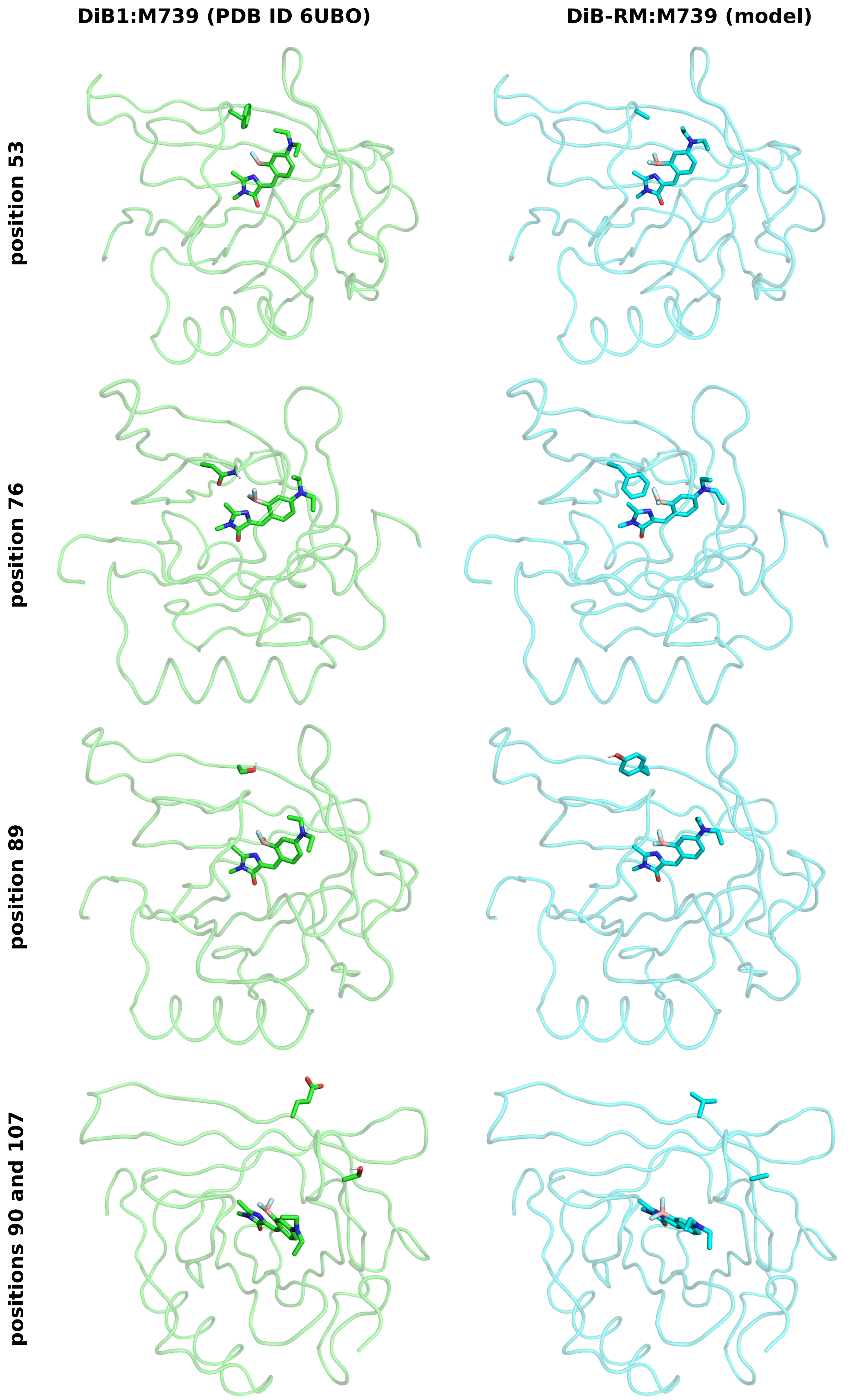

Supplement: S8 Fig — (TIF) [file pcbi.1009555.s009.tif]
